# Folate Intake and the Risk of Breast Cancer: A Dose-Response Meta-Analysis of Prospective Studies

**DOI:** 10.1371/journal.pone.0100044

**Published:** 2014-06-16

**Authors:** Yu-Fei Zhang, Wei-Wu Shi, Hong-Fang Gao, Li Zhou, An-Ji Hou, Yu-Hao Zhou

**Affiliations:** 1 Department of Oncology, Shanghai Seventh People’s Hospital, Shanghai, China; 2 Medical Research Center, Taizhou Hospital of Zhejiang Province, Wenzhou Medical College, Linhai, Zhejiang, China; 3 Department of Rehabilitation Institute, Shanghai Seventh People’s Hospital, Shanghai, China; Cardiff University, United Kingdom

## Abstract

**Background:**

Previous observational studies regarding the existence of an association between folate intake and the risk of breast cancer have been inconsistent. This study aimed to summarize the evidence regarding this relationship using a dose-response meta-analytic approach.

**Methodology and Principal Findings:**

We performed electronic searches of the PubMed, EmBase, and Cochrane Library databases to identify studies published through June 2013. Only prospective observational studies that reported breast cancer effect estimates with 95% confidence intervals (CIs) for more than 2 folate intake categories were included. We excluded traditional case-control studies because of possible bias from various confounding factors. Overall, we included 14 prospective studies that reported data on 677,858 individuals. Folate intake had little effect on the breast cancer risk (relative risk (RR) for highest versus lowest category = 0.97; 95% CI, 0.90–1.05; P = 0.451). Dose-response meta-analysis also suggested that a 100 µg/day increase in folate intake had no significant effect on the risk of breast cancer (RR = 0.99; 95% CI, 0.98–1.01; P = 0.361). Furthermore, we used restricted cubic splines to evaluate the nonlinear relationship between folate intake and the risk of breast cancer, and discovered a potential J-shaped correlation between folate intake and breast cancer risk (P = 0.007) and revealed that a daily folate intake of 200–320 µg was associated with a lower breast cancer risk; however, the breast cancer risk increased significantly with a daily folate intake >400 µg.

**Conclusion/Significance:**

Our study revealed that folate intake had little or no effect on the risk of breast cancer; moreover, a dose-response meta-analysis suggested a J-shaped association between folate intake and breast cancer.

## Introduction

Given its role as a modulator of DNA synthesis, repair, and methylation, folate was hypothesized to reduce the risk of breast cancer [1]. The importance of these processes in cell growth and development led to investigations of the consequences of either low or high folate intake on cancer development [2]. The results of several studies suggested that increased folate intake was associated with an increased prostate cancer risk [3] but significantly reduced risks of esophageal, stomach, pancreatic [4], and colorectal cancers [5]. However, data regarding the subsequent effects of folate intake on breast cancer are limited and inconclusive.

A meta-analysis [6] based on 9 prospective studies and 14 case-control studies suggested no clear association between the folate intake or blood folate levels and the risk of breast cancer; however, folate appeared to significantly counteract the increased risk of breast cancer associated with moderate or high levels of alcohol consumption. It is particularly important to clarify the optimal daily folate intake level with respect to the general population, as this level has yet not been definitively determined. Furthermore, additional unanswered questions remain, including whether associations differ according to follow-up duration, and alcohol intake.

Folate intake has been studied in numerous prospective studies of primary breast cancer prevention and, in the present study, we attempted a large-scale examination of these available prospective studies to update the results and determine the association between folate intake and the risk of breast cancer. Furthermore, we performed a dose-response meta-analysis to quantitatively determine the optimal folate intake level with respect to the general population.

## Methods

### Data Sources, Search Strategy, and Selection Criteria

This review was conducted and reported according to the Preferred Reporting Items for Systematic Reviews and Meta-Analysis Statement [7] issued in 2009 (Checklist S1).

Any prospective study that examined the relationship between folate intake and breast cancer was eligible for inclusion in our study, and no restrictions were placed on the publication language or status (published, in press, or in progress). We searched the PubMed, EmBase, and Cochrane Library electronic databases for articles published through June 2013 using the following search terms (“folate” OR “folic acid”) AND (“cancer” OR “neoplasm” OR “carcinoma”) AND (“cohort” OR “cohort studies” OR “nest case-control studies”). We also conducted manual searches of the reference lists from all relevant original and review articles to identify additional eligible studies. The medical subject headings, methods, patient populations, designs, exposures, and outcome variables of these articles were used to identify the relevant studies. Two of the authors (HFG and YFZ) conducted this literature search independently, according to a standardized approach. Any inconsistencies between the 2 authors were settled by the primary author (YHZ) until a consensus was reached. Studies were eligible for inclusion if the following criteria were met: (1) the study had a prospective design (prospective cohort or nested prospective case-control study); (2) the study investigated the association between folate intake and the risk of breast cancer; and (3) the authors reported the effect estimates (risk ratio [RR], hazard ratio [HR], or odds ratio [OR]) and 95% confidence intervals (CIs) for comparisons of highest and lowest category folate intake and included >2 folate intake categories. We excluded all case-control studies because various confounding factors could have biased the results.

### Data Collection and Quality Assessment

The collected data included the first author’s or study group’s name, publication year, country, study design, folate exposure assessment, sample size, age at baseline, follow-up duration, effect estimate and 95% CI, comparison categories, and covariates in the fully adjusted model. We also extracted the numbers of cases/persons or person-years, the effects of the different exposure categories, and the 95% CIs. For studies that reported several multivariable adjusted-effect estimates, we selected the effect estimate that had been maximally adjusted with respect to potential confounders.

The highly comprehensive Newcastle-Ottawa Scale (NOS) [8], which has been partially validated for quality evaluations of observational studies in meta-analyses, was used to evaluate the methodological quality [9]. The NOS is based on the following 3 subscales: selection (4 items), comparability (1 item), and outcome (3 items). A “star system” (range, 0–9) was developed for assessment purposes (Table S1). The data extraction and quality assessments were conducted independently by 2 authors (HFG and LZ). The information was examined and adjudicated independently by an additional author (YHZ) with reference to the original studies.

### Statistical Analysis

We examined the relationship between folate intake and the risk of breast cancer on the basis of the effect estimates (RR or HR) and 95% CI published in each study. We first used the random-effects model [10], [11] to calculate the summary RRs and 95% CIs for highest versus lowest category folate intake levels. Second, we transformed category-specific risk estimates into RR estimates associated with an increase in folate intake of 100 µg/day by using the method of generalized least squares for trend estimation [12]. These estimates were calculated by assuming a linear relationship between the natural logarithm of RR and increasing folate intake. The value assigned to each folate category was the mid-point for closed categories and the median for open categories (assuming a normal distribution for folate intake). We combined the RRs for each 100 µg/day increase in folate intake by using the results of a random-effect meta-analysis [10]. Third, We conducted a dose-response random-effects meta-analysis of the correlated natural logs of the RRs or HRs across all folate intake categories [12], [13]. To derive the dose-response curve, we modeled folate using restricted cubic splines with 3 knots at fixed distribution percentiles of 10%, 50%, and 90% [12]. This method required knowledge of the distributions of cases and persons or person-years and effect estimates (RRs or HRs) along with the variance estimates for at least 3 quantitative exposure categories.

Inter-study heterogeneity was investigated with the Q statistic, and we considered P-values<0.10 as indicative of significant heterogeneity [14], [15]. Breast cancer subgroup analyses were conducted on the basis of the country, study design, sample size, effect estimate (HR or RR), follow-up duration, adjusted alcohol intake, and alcohol intake. We also performed a sensitivity analysis by removing each individual study from the meta-analysis.

Several methods were used to evaluate the potential publication bias. Funnel plots for the risk of breast cancer were visually inspected. The Egger [16] and Begg [17] tests were also used to statistically assess the publication bias with respect to the breast cancer incidence. All reported P values were 2-sided, and P values<0.05 were considered statistically significant for all included studies. STATA software (version 12.0; Stata Corporation, College Station, TX, USA) was used to perform the statistical analyses.

## Results

**Figure 1:**
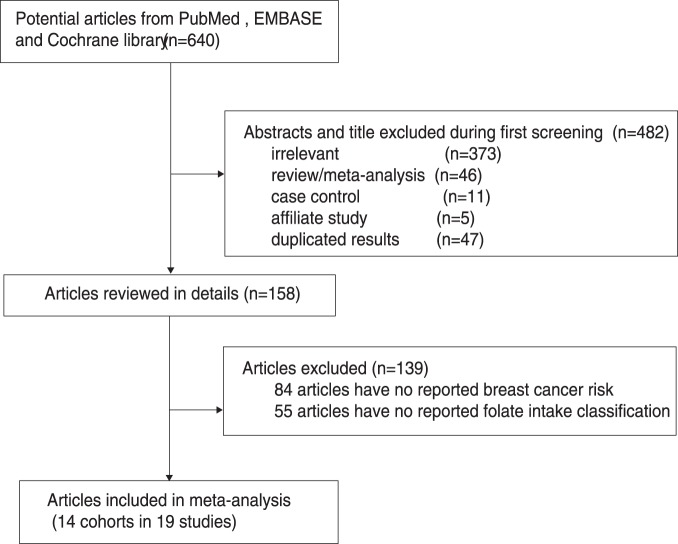
Flow diagram of the literature search and studies selection process.

The results of the study-selection process are shown in Figure 1. We identified 640 articles during our initial electronic search, of which 482 were excluded as duplicates or irrelevant studies. A total of 158 potentially eligible studies were thus selected. After a detailed evaluation, 14 cohorts from 19 studies [18]–[36] were selected for the final meta-analysis. A manual search of the reference lists of these studies did not yield any new eligible studies. The general characteristics of the included studies are presented in Table 1.

**Table 1:** Baseline characteristic of studies included in the systematic review and meta-analysis.

Study	Country	Studydesign	Assessmentof exposure	Samplesize	Age atbaseline	Effectestimate	Comparisoncategories∧	Follow-up(year)	Covariates in fullyadjusted model
Zhang S 1999[18]–[19]	US	Cohort	FFQ	88818	30–55	RR	>600 vs 150–299 ug/d	16.0	Age, length of follow-up,total energy intake, parity,age at first birth, history of BC inmother or a sister, history ofbenign BC, alcohol intake,BMI at age 18 years, weightchange from age 18 years, height,age at menopause, HRT use, andbeta-carotene
Rohan TE 2000[20]	Canada	Cohort	FFQ	56837	NG	RR	>354.28 vs <224.78 ug/d	8.0	Energy intake, age, age at menarche,number of live births, menopausalstatus, family history of BC in afirst-degree relative, practice ofbreast self-examination, alcoholconsumption, randomization group,and study center.
Sellers TA 2001[21]–[22]	US	Cohort	FFQ	34387	62.0	RR	>294 vs <172 ug/d	12.0	Age, education, family history of BC, age at menarche, age at menopause, oral contraceptive use, HRT, parity, age at first birth, BMI, waist-to-hip ratio, height, BMI at age 18, alcohol, smoking, PA, and other B vitamins
Feigelson HS 2003[23]–[24]	US	Cohort	FFQ	66561	40–87	RR	>918.9 vs <192.2 ug/d	14.0	Age, includes ethanol, dietary folate,methionine, multivitamin use, race,education, first-degree family historyof BC, history of breast lump,mammographic history, HRT use,parity and age at first birth, age atmenopause, age at menarche, PA,BMI, adult weight gain, and energy
Baglietto L 2005[25]–[26]	Anglo-Australian	Cohort	FFQ	17447	40–69	HR	400 vs 200 ug/d	13.8	Total energy and folate intake andfitted as linear variable in Cox’sproportional hazard model withage as time metric.
Stolzenberg-SolomonRZ 2006 [27]	US	Cohort	FFQ	25400	55–74	HR	>853.0 vs <335.5 ug/d	9.8	Energy, education, HRT,mammography screening history,birth control pill use, history ofbenign breast disease, family historyof BC, age at menarche, age atmenopause, and age at first birthand number of live births.
Lajous M 2006[28]	French	Cohort	FFQ	62739	40–65	RR	522 vs 296 ug/d	9.0	Age, two-year follow-up period,region of residence, years ofeducation, family BC, history ofbenign breast disease, age atmenarche, parity, breastfeeding,years since last use of oralcontraceptives, age at menopause,years of HRT use, regularmammographic evaluation, heightin cm, BMI, vitamin supplement use,alcohol intake and PA.
Tjønneland A 2006[29]–[30]	Denmark	Nest casecontrol	FFQ	24697	50–64	RR	>400 vs <300 ug/d	4.7	Vitamin C, total energy, schooleducation, BMI, parous/nulliparousand number of births, age at birth offirst child, history of benign breasttumour surgery.
Ericson U 2007[31]	Sweden	Cohort	FFQ	11699	>50	HR	456 vs 160 ug/d	9.5	Weight, height, leisure-time PA,household work, smoking,socioeconomic status, age atmenopause, HRT, and alcohol intake.
Larsson SC 2008[32]	Sweden	Cohort	FFQ	61433	40–70	RR	>277 vs <200 ug/d	17.4	Age, education, body mass index,height, parity, age at first birth,age at menarche, age at menopause,use HRT, family history of breastcancer, history of benign breastdisease, and intakes of alcoholand total energy
Lin J 2008[33]	US	Nest case control	FFQ	28345	>45	RR	>582 vs <263.9 ug/d	11.0	Matching variables, age, randomizedtreatment assignment, BMI, familyhistory of BC in a first-degreerelative, history of benign breastdisease, smoking, PA, alcoholconsumption, age at menarche,age at menopause, parity, andage at first birth.
Maruti SS 2009[34]	US	Cohort	FFQ	35023	50–76	RR	541 vs 210 ug/d	5.0	Age, race, family history of BC,mammography within 2 ypreceding baseline, history of breastbiopsy, age at menarche, age at firstbirth, age at menopause, years ofHRT use, BMI, height, total PA,and alcohol intake in the past year,total energy intake.
Duffy CM 2009[35]	US	Cohort	FFQ	88530	50–79	HR	>642 vs <227.6 ug/d	5.5	Tobacco consumption, BMI, historyof breast biopsy, number ofpregnancies, ever breast fed, familyhistory, previous combined HRTuse, age at menarche, age atmenopause.
Shrubsole MJ 2011[36]	China	Cohort	FFQ	74942	40–70	HR	404 vs 194 ug/d	12.0	Age at baseline, age at menarche,parity, age at first livebirth,educational attainment, PA, use of aB vitamin supplement, height, andtotal daily intakes of energy,vegetables, fat, and menopausalstatus

*BC: breast cancer; BMI: body mass index; FFQ: food frequency questionnaire; HRT: hormone replacement therapy; PA: physical activity.

∧The detailes of folate intake dose were listed in Table S2.

Of the 14 included cohorts (for a total of 677,858 individuals), 12 cohorts were from 16 prospective cohort studies [18]–[28], [31], [32], [34], [36] and 2 cohorts were from 3 nested case-control studies [29], [30], [33]. The participant follow-up period ranged 4.7–17.4 years and the number of individuals per study ranged 11,699–88,818. Seven cohorts [18], [19], [21]–[24], [27], [33]–[35] were based in the US, 5 [25], [26], [28]–[32] in Europe, 1 [20] in Canada, and 1 [36] in China. The study quality was assessed according to the NOS [8] (Table S1). Herein, we considered a study with a score ≥7 to be high quality. Overall, 3 cohorts [20], [23], [24], [31] had scores of 9, 9 cohorts [18], [19], [21], had scores of 8, 1 cohort [33] had a score of 7, and 1 cohort [35] had a score of 6.

**Figure 2:**
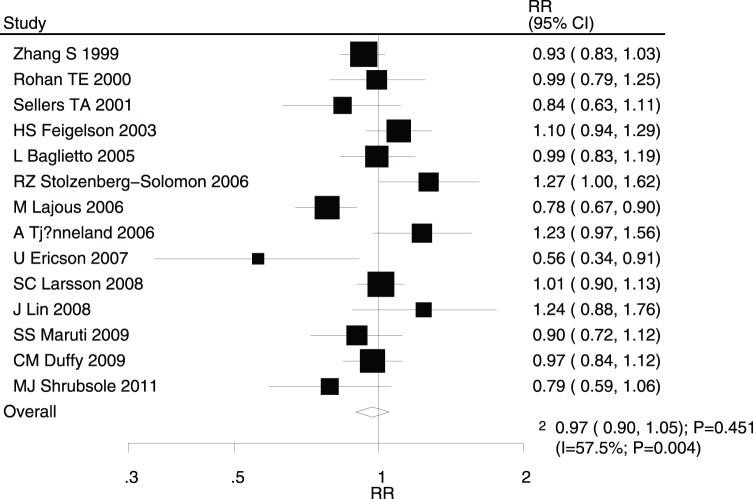
Relative risk estimates of breast cancer for highest versus lowest folate intake category.

**Figure 3:**
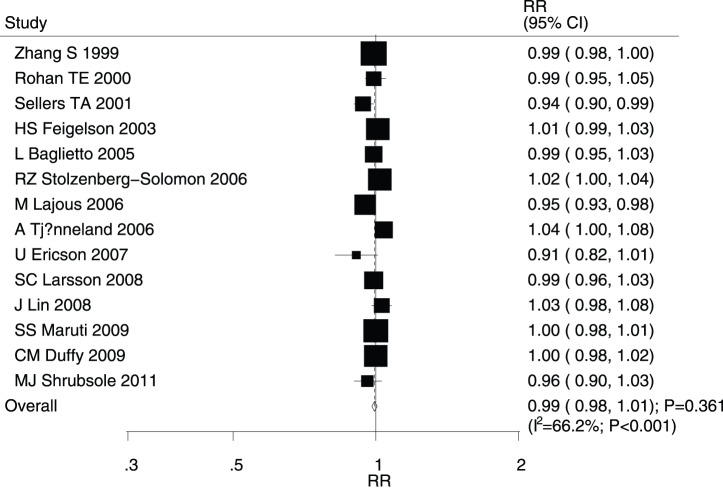
Dose-response meta-analysis for per 100 µg/day increment in folate intake for breast cancer.

After pooling the included studies, the summary RR revealed that folate intake was not associated with breast cancer (RR for highest versus lowest category = 0.97; 95% CI, 0.90–1.05; P = 0.451; Figure 2); however, evidence of potentially significant heterogeneity was noted (I^2^ = 57.5%; P = 0.004). Consequently, a sensitivity analysis was conducted and after sequentially excluding each study from the pooled analysis, the conclusion was not found to be affected by the exclusion of any specific study. The dose-response meta-analysis findings did not suggest any association between the risk of breast cancer and a 100 µg/day increase in folate intake (RR, 0.99; 95% CI, 0.98–1.01; P = 0.361; Figure 3), with substantial heterogeneity across studies (I^2^ = 66.2%; P<0.001).

**Figure 4:**
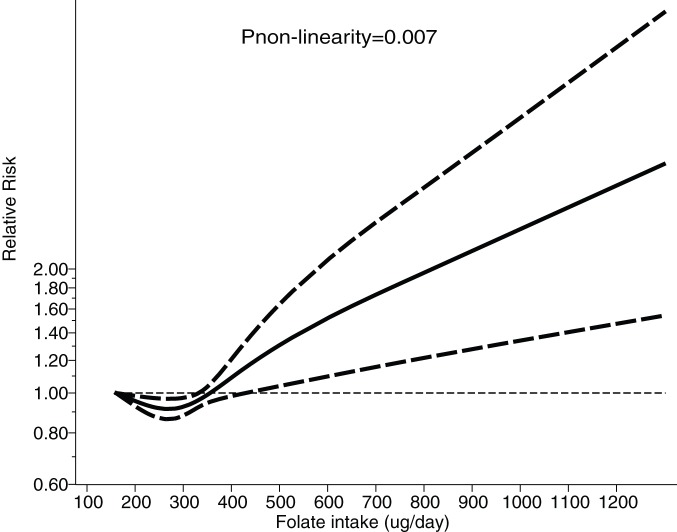
Dose-response relations between folate intake and relative risks of breast cancer.

All studies were included in the dose-response meta-analysis of the relationship between folate intake and the risk of breast cancer. As shown in Figure 4 and by the P-value for nonlinearity (P = 0.007), we discovered evidence of a nonlinear relationship between folate intake and the risk of breast cancer. A daily folate intake of 200–320 µg was associated with reduced the risk of breast cancer. This potential preventive effect was refuted up to a daily folate intake of 320 µg, and a small effect and borderline statistical significance was maintained up to a daily folate consumption of 400 µg; however, daily folate intake levels >400 µg was associated with increased the risk of breast cancer.

**Figure 5:**
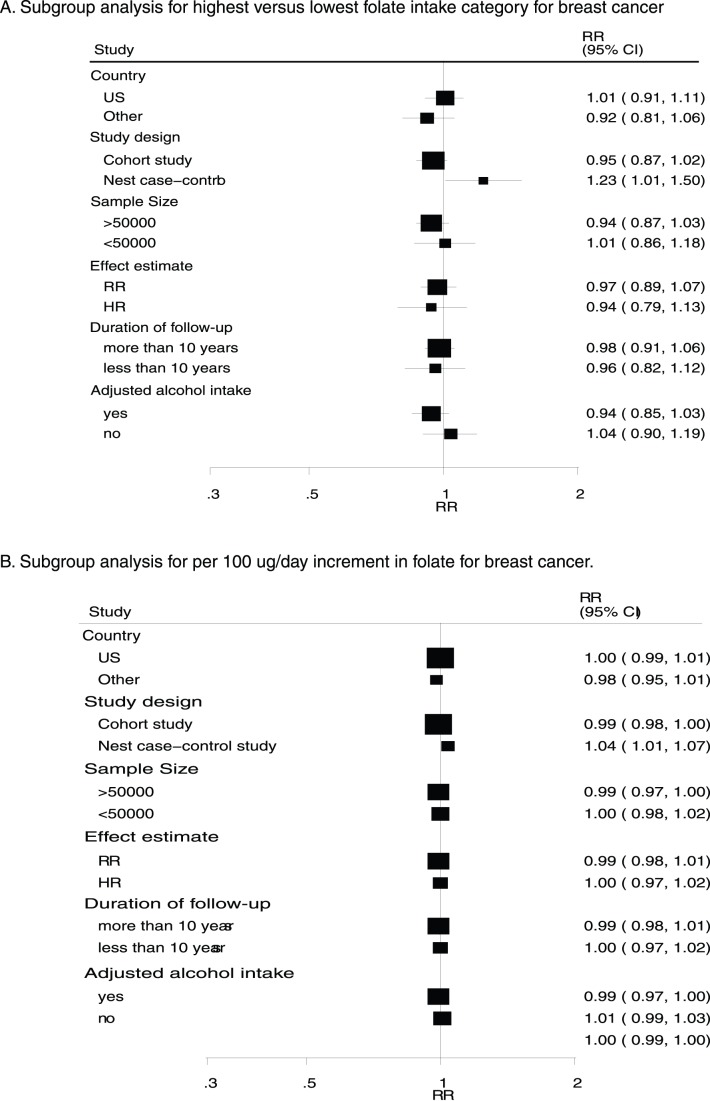
Subgroup analysis for breast cancer.

A heterogeneity assessment of this analysis yielded a P-value<0.10. Accordingly, we conducted subgroup analyses to minimize heterogeneity among the included studies. Overall, we noted that folate intake level was associated with a reduced breast cancer risk if the patients had a daily alcohol intake >10 g (RR for highest versus lowest category = 0.64; 95% CI: 0.43–0.97). Conversely, folate intake was associated with an increased risk of breast cancer when nested case-control studies were included (RR for highest versus lowest category = 1.23; 95% CI: 1.01–1.50; Figure 5). Furthermore, Subgroup analysis revealed that a 100 µg/day increment in folate intake was also associated with increased breast cancer risk (RR = 1.04; 95% CI: 1.01–1.07; Figure 5) when nested case-control studies were included. No other significant differences were identified with respect to the effects of increased folate intake in association with additional factors.

**Figure 6:**
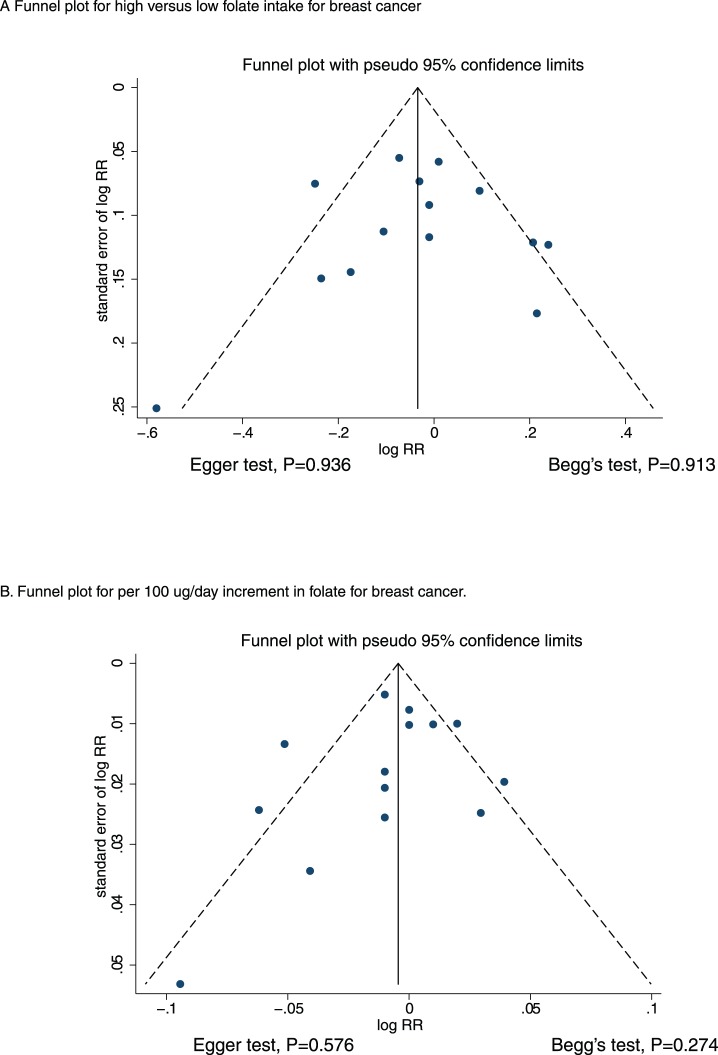
Funnel plot for breast cancer.

A review of the funnel plots did not rule out the possibility of publication bias with respect to breast cancer (Figure 6). However, the Egger [16] and Begg [17] test results did not show any evidence of publication bias, P = 0.936 and P = 0.913 for highest versus lowest category folate intake, respectively, and P = 0.576 and P = 0.274 for a 100 µg/day increment in folate intake, respectively.

## Discussion

The current meta-analysis evaluated prospective studies to explore all possible correlations between folate intake and the risk of breast cancer. This large quantitative study included 677,858 individuals from 12 prospective cohort studies and 2 nested case-control studies with a broad population range. The findings from our current meta-analysis suggest that there are no effects of increased folate intake on the incidence of breast cancer.

A previous meta-analysis [6] of observational studies (cohort studies, nested case-control studies, and case-control studies) suggested that folate intake or blood folate levels had no effect on the risk of breast cancer and that adequate folate intake significantly reduced the risk of breast cancer in individuals with moderate or high levels of alcohol consumption. However, the various confounding factors present in case-control studies could lend bias to the results. Furthermore, the cutoff points for the folate intake categories differed among the studies. Finally, we also reported the association between folate intake and breast cancer in some specific subsets. Another meta-analysis of randomized controlled trials that was published by the B-Vitamin Treatment Trialists’ Collaboration [37] suggested that folic acid supplementation did not affect the risk of breast cancer. An inherent limitation of the earlier meta-analysis was that the included trials had been designed to evaluate the effects of folic acid supplementation on cardiovascular or other outcomes rather than cancer-related outcomes; additionally, the results had been derived from very few cases and should thus have been considered preliminary results. Furthermore, the follow-up duration was insufficiently long to demonstrate a clinical benefit and thus always yielded broad confidence intervals (i.e., no statistically significant difference). Thus far, no study has confirmed the association between folate intake and the risk of breast cancer. Therefore, we conducted a dose-response meta-analysis of the existing prospective studies in order to identify the optimal folate intake dose.

Most of our findings agreed with those of a recently published large cohort study conducted in the US [18]; that prospective study included 88,818 individuals and found that participants who consumed >600 µg of folate per day exhibited a 7% decrease in the risk of breast cancer when compared with individuals who consumed 150–299 µg of folate per day, although this decrease was not statistically significant. Furthermore, adequate folate intake might reduce the excess risk of breast cancer associated with alcohol consumption. Rohan et al. [20] also suggested that increased folate intake did not affect the overall risk of breast cancer but significantly reduced the risk of breast cancer in participants with a daily alcohol consumption level >14 g. Our current study also indicated that increased folate intake level had no significant effect on the overall risk of breast cancer but that folate might play an important role with respect to the risk of breast cancer in participants with high alcohol consumption levels. Possible reasons for these findings might include the different folate intake category cutoff points among the studies as well as data collection methods that only compared the breast cancer risks at highest and lowest category folate intake levels. Furthermore, because folate could potentially promote tumor cell growth, high blood folate levels might be associated with an increased risk of breast cancer [38], [39].

No significant difference was observed between increased folate intake and the risk of breast cancer. However, several studies that were included in our meta-analysis reported inconsistent results. Lajous et al. [28] indicated that folate intake level was associated with a reduced risk of breast cancer (highest versus lowest category). Furthermore, Ericson et al. [31] also suggested that folate intake was associated with reduced the risk of breast cancer by 44% (highest versus lowest category). However, those 2 cohorts had low power with which to detect the interaction between folate and alcohol intake and, moreover, most of the women in those cohorts consumed alcohol whereas few used folate supplements. These 2 factors could possibly explain why those studies found that folate exhibited an overall protective effect against breast cancer.

In restricted cubic splines analysis, we identified a potential nonlinear relationship between folate intake and breast cancer suggestive of an association between a daily folate intake of 200–320 µg and a lower breast cancer risk; however, a daily folate intake >400 µg appeared to significantly increase the risk of breast cancer. A possible explanation for this finding is that folate normally acts as a modulator of DNA synthesis, repair, and methylation [1] but at a daily folate intake >400 µg levels, folate might affect endothelial function and support cell growth [40].

A subgroup analysis suggested that increased folate intake was associated with reduced the risk of breast cancer in patients with a daily alcohol consumption level >10 g. Several studies [18]–[20] included in our analysis reported results consistent with this finding. Furthermore, folate intake was associated with increased the risk of breast cancer when we pooled the nested case-control studies. However, this conclusion might have been unreliable because smaller cohorts were included in that subset. Therefore, we simply reported the relative result and thus provided a synthetic and comprehensive review.

Two strengths of our study should be highlighted. First, only prospective studies were included; this restriction should have eliminated selection and recall biases, which might have been of concern with regard to the retrospective case-control studies. Second, the dose-response analysis included a broad folate intake range, thus allowing an accurate assessment of the dose-response relationship between folate intake and the risk of breast cancer.

The limitations of our study were as follows: (1) publication bias is an inevitable problem in meta-analyses of published studies; (2) data regarding breast cancer in premenopausal or postmenopausal women were unavailable; and (3) the analysis used pooled data (individual data were not available), which restricted us from performing a more detailed relevant analysis and obtaining more comprehensive results.

The results of this study suggest that increased folate intake has no significant effect on the risk of breast cancer. According to our dose-response meta-analysis, a daily folate intake of 200–320 µg appeared to associate with a lower risk of breast cancer; in contrast, increased breast cancer risk was associated with a daily folate intake >400 µg/d. Future studies should focus on specific populations in order to analyze primary breast cancer prevention.

## Supporting Information

Table S1 **Quality scores of prospective cohort studies using Newcastle-Ottawa Scale.**
(DOC)

Table S2 **Daily intakes of folate and risk of breast cancer data in included studies.**
(DOC)

Checklist S1 **PRISMA Checklist.**
(DOC)
