# Supplementary material for: Folate Intake and the Risk of Breast Cancer: A Dose-Response Meta-Analysis of Prospective Studies
Source: PLoS One. 2014 Jun 16;9(6):e100044. doi: 10.1371/journal.pone.0100044 (PMC4059748; doi:10.1371/journal.pone.0100044)
Supplement: Table S1 — Quality scores of prospective cohort studies using Newcastle-Ottawa Scale. (DOC) [file pone.0100044.s001.doc]

Table S1. Quality scores of prospective cohort studies using Newcastle-Ottawa Scale.

| Study | Selection | | | | Comparability | Outcome | | | NOS |
| --- | --- | --- | --- | --- | --- | --- | --- | --- | --- |
|  | Representativeness of the exposed cohort | Selection of the non exposed cohort | Ascertainment  of folate intake | Demonstration that outcomes was not present at start of study | Comparability on the basis of the design or analysis | Assessment of outcome | Adequate follow-up duration | Adequate follow-up rate | Overall score |
| Zhang S^18-19^ 1999 | 1 | 1 | 1 | 1 | 2 | 1 | 1 | 0 | 8 |
| Rohan TE^20^ 2000 | 1 | 1 | 1 | 1 | 2 | 1 | 1 | 1 | 9 |
| Sellers TA^21-22^ 2001 | 0 | 1 | 1 | 1 | 2 | 1 | 1 | 1 | 8 |
| HS Feigelson^23-24^ 2003 | 1 | 1 | 1 | 1 | 2 | 1 | 1 | 1 | 9 |
| L Baglietto^25-26^ 2005 | 1 | 1 | 1 | 1 | 1 | 1 | 1 | 1 | 8 |
| RZ Stolzenberg-Solomon^27^ 2006 | 0 | 1 | 1 | 1 | 2 | 1 | 1 | 1 | 8 |
| M Lajous^28^ 2006 | 1 | 1 | 0 | 1 | 2 | 1 | 1 | 1 | 8 |
| A Tjønneland^29-30^ 2006 | 1 | 1 | 1 | 1 | 2 | 1 | 0 | 1 | 8 |
| U Ericson^31^ 2007 | 1 | 1 | 1 | 1 | 2 | 1 | 1 | 1 | 9 |
| SC Larsson^32^ 2008 | 1 | 1 | 1 | 1 | 2 | 1 | 1 | 0 | 8 |
| J Lin^33^ 2008 | 0 | 1 | 1 | 0 | 2 | 1 | 1 | 1 | 7 |
| SS Maruti^34^ 2009 | 1 | 1 | 1 | 1 | 2 | 1 | 0 | 1 | 8 |
| CM Duffy^35^ 2009 | 0 | 1 | 1 | 0 | 2 | 1 | 0 | 1 | 6 |
| MJ Shrubsole^36^ 2011 | 1 | 1 | 1 | 0 | 2 | 1 | 1 | 1 | 8 |
