# Supplementary material for: Folate Intake and the Risk of Breast Cancer: A Dose-Response Meta-Analysis of Prospective Studies
Source: PLoS One. 2014 Jun 16;9(6):e100044. doi: 10.1371/journal.pone.0100044 (PMC4059748; doi:10.1371/journal.pone.0100044)
Supplement: Table S2 — Daily intakes of folate and risk of breast cancer data in included studies. (DOC) [file pone.0100044.s002.doc]

Table S2. Daily intakes of folate and risk of breast cancer data in included studies

| **Study** | **Country** | **Folate dose** **(ug per day)** | **Breast cancer cases** | **Number of individuals or person-years** | **Relative risk and 95%CI** |
| --- | --- | --- | --- | --- | --- |
| Zhang S 1999 | US | 223 | 1411 | 552975 | 1.00 |
|  |  | 357 | 953 | 356862 | 0.93 (0.85-1.02) |
|  |  | 527 | 535 | 183488 | 1.00 (0.91-1.11) |
|  |  | 814 | 472 | 184204 | 0.93 (0.83-1.03) |
| Rohan TE 2000 | Canada | 179.8 | 258 | 113868 | 1.00 |
|  |  | 245.8 | 263 | 113501 | 0.98 (0.78-1.23) |
|  |  | 285.9 | 266 | 111799 | 1.01 (0.80-1.28) |
|  |  | 329.6 | 264 | 113626 | 0.95 (0.76-1.20) |
|  |  | 425.1 | 285 | 113397 | 0.99 (0.79-1.25) |
| Sellers TA 2001 | Iowa | 167.4 | 163 | 37131 | 1.00 |
|  |  | 228.5 | 339 | 74785 | 0.93 (0.76-1.13) |
|  |  | 311 | 299 | 74905 | 0.76 (0.65-0.92) |
|  |  | 526.5 | 785 | 185821 | 0.84 (0.63-1.11) |
| HS Feigelson 2003 | US | 157.4 | 307 | 16261 | 1.00 |
|  |  | 264.8 | 317 | 16335 | 0.99 (0.85-1.17) |
|  |  | 461.8 | 331 | 16216 | 1.04 (0.89-1.22) |
|  |  | 754.6 | 348 | 16446 | 1.10 (0.94-1.29) |
| L Baglietto 2005 | Anglo-Australian | 224 | 238 | 5189 | 1.00 |
|  |  | 286 | 198 | 5189 | 0.80 (0.66-0.96) |
|  |  | 340 | 241 | 5189 | 0.93 (0.78-1.12) |
|  |  | 422 | 259 | 5189 | 0.99 (0.83-1.19) |
| RZ Stolzenberg-Solomon 2006 | US | 262.7 | 115 | 25868 | 1.00 |
|  |  | 416.9 | 134 | 26645 | 1.05 (0.82-1.35) |
|  |  | 649.8 | 153 | 24767 | 1.32 (1.03-1.68) |
|  |  | 773.9 | 130 | 25292 | 1.03 (0.80-1.33) |
|  |  | 1153.6 | 159 | 24689 | 1.27 (1.00-1.62) |
| M Lajous 2006 | French | 296 | 395 | 77779 | 1.00 |
|  |  | 350 | 355 | 80108 | 0.85 (0.73-0.98) |
|  |  | 392 | 366 | 82379 | 0.85 (0.73-0.98) |
|  |  | 440 | 347 | 83957 | 0.79 (0.68-0.91) |
|  |  | 522 | 349 | 86091 | 0.78 (0.67-0.90) |
| A Tjønneland 2006 | Denmark | 225 | 116 | 236 | 1.00 |
|  |  | 325 | 59 | 131 | 1.14 (0.95-1.37) |
|  |  | 375 | 62 | 127 | 1.13 (0.93-1.38) |
|  |  | 500 | 140 | 261 | 1.23 (0.97-1.56) |
| U Ericson 2007 | Sweden | 160 | 71 | 21587 | 1.00 |
|  |  | 204 | 78 | 22124 | 0.99 (0.68-1.44) |
|  |  | 236 | 85 | 22104 | 1.01 (0.68-1.50) |
|  |  | 284 | 84 | 22691 | 0.94 (0.62-1.43) |
|  |  | 456 | 74 | 22418 | 0.56 (0.34-0.91) |
| SC Larsson 2008 | Sweden | 177 | 577 | 211726 | 1.00 |
|  |  | 212 | 560 | 213590 | 0.94 (0.84-1.06) |
|  |  | 235 | 596 | 215023 | 0.99 (0.88-1.11) |
|  |  | 260 | 577 | 214797 | 0.93 (0.83-1.05) |
|  |  | 315 | 642 | 216029 | 1.01 (0.90-1.13) |
| J Lin 2008 | US | 211.1 | 177 | 342 | 1.00 |
|  |  | 289 | 138 | 303 | 0.81 (0.57-1.15) |
|  |  | 347.5 | 154 | 319 | 0.87 (0.62-1.23) |
|  |  | 481.5 | 168 | 333 | 1.00 (0.71-1.40) |
|  |  | 698.4 | 191 | 356 | 1.24 (0.88-1.76) |
| SS Maruti 2009 | US | 325 | 153 | 8755 | 1.00 |
|  |  | 604 | 91 | 8756 | 0.92 (0.70-1.20) |
|  |  | 960 | 193 | 8756 | 1.02 (0.82-1.27) |
|  |  | 1302 | 222 | 8756 | 0.90 (0.72-1.12) |
| CM Duffy 2009 | US | 182.1 | 340 | 19686 | 1.00 |
|  |  | 319.3 | 411 | 19697 | 1.01 (0.87-1.16) |
|  |  | 526.5 | 428 | 19686 | 1.05 (0.91-1.22) |
|  |  | 770.4 | 420 | 19687 | 0.97 (0.84-1.12) |
| MJ Shrubsole 2011 | China | 194 | 146 | 14988 | 1.00 |
|  |  | 243 | 142 | 14989 | 0.90 (0.71-1.15) |
|  |  | 282 | 143 | 14988 | 0.89 (0.69-1.15) |
|  |  | 328 | 160 | 14989 | 0.99 (0.76-1.28) |
|  |  | 404 | 127 | 14988 | 0.79 (0.59-1.06) |
